# Nonpalpable testicular pure seminoma with elevated serum alpha-fetoprotein presenting with retroperitoneal metastasis: a case report

**DOI:** 10.1186/s13256-016-0906-7

**Published:** 2016-05-05

**Authors:** Shoichiro Iwatsuki, Taku Naiki, Noriyasu Kawai, Toshiki Etani, Keitaro Iida, Ryosuke Ando, Takashi Nagai, Atsushi Okada, Keiichi Tozawa, Yosuke Sugiyama, Takahiro Yasui

**Affiliations:** 1 Department of Nephro-urology, Graduate School of Medical Sciences, Nagoya City University, 1, Kawasumi, Mizuho-cho, Mizuho-ku, 467-8601 Nagoya, Japan; 2 Department of Pharmacy, Nagoya City University Hospital, Nagoya, Japan

**Keywords:** Alpha-fetoprotein, DUPAN-2, Testicular tumor, Retroperitoneal lymph node dissection, Seminoma, Burned-out tumor, Case report

## Abstract

**Background:**

Patients with a primary pure seminoma in the testis who have elevated serum alpha-fetoprotein are rare and should be treated as patients with nonseminomatous germ cell tumors. However, nonpalpable testicular tumors in this condition have never been reported. We describe a case of nonpalpable pure testicular seminoma with elevated serum alpha-fetoprotein presenting retroperitoneal metastasis.

**Case presentation:**

A 29-year-old Asian man was referred to our hospital with right flank pain. Computed tomography showed a mass located between his aorta and inferior vena cava, but a testicular tumor was not detected. His serum levels of lactate dehydrogenase, alpha-fetoprotein, and DUPAN-2 were high. Although no tumor or nodule was palpable in his testis, ultrasonography revealed multiple low echoic lesions in his right testicular parenchyma. He was diagnosed with right testicular cancer with retroperitoneal lymph node metastasis and underwent right high orchiectomy. A pathological examination revealed pure seminoma and no nonseminomatous components were found in the specimen. Three courses of induction systemic chemotherapy (cisplatin, etoposide, and bleomycin) normalized his serum alpha-fetoprotein and DUPAN-2 levels. Three additional courses of chemotherapy (etoposide and bleomycin) were performed, and treatment was completed with laparoscopic retroperitoneal lymph node dissection. Pathology of the dissected specimen showed fibrous and necrotic tissue with no viable cells. He is alive without recurrence 54 months after orchiectomy.

**Conclusions:**

We report a case of pure testicular seminoma with elevated serum alpha-fetoprotein and DUPAN-2 presenting retroperitoneal metastasis. We recommend an ultrasound examination of bilateral testes when large retroperitoneal tumors are detected in young men, even if a mass is not palpable in the scrotum.

## Background

Testicular cancers are categorized into seminomatous and non-seminomatous germ cell tumors (NSGCTs), each with a 50 % incidence. Distinguishing NSGCTs from seminomas is important because the therapeutic strategy is different. Many patients with NSGCT have elevated serum tumor markers, such as lactate dehydrogenase (LDH), alpha-fetoprotein (AFP), and human chorionic gonadotropin (hCG). Since seminoma does not induce AFP production, the guidelines recommend that cases with increased AFP levels be treated as cases of NSGCT [1, 2]. Because, there is a possibility that a hidden focus of NSGCTs like yolk sac tumor is somewhere present. However, in the literature, few cases have been described of patients with histologically pure seminoma presenting with elevated serum AFP levels [3–5].

We report a case of a 29-year-old Asian man with nonpalpable testicular cancer presenting retroperitoneal lymph node metastasis. Although his testicular histology showed pure seminoma at orchiectomy, preoperative serum AFP levels were elevated.

## Case presentation

**Fig. 1:**
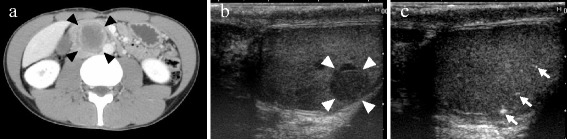
**a** Computed tomography shows a retroperitoneal tumor located between the aorta and the inferior vena cava. The size of the tumor (*arrowheads*) was 52×36×31 mm, and the testicular tumor was not detected in computed tomography. **b** Ultrasound examination revealed a tiny low echoic tumor (*arrowheads*) and a low echoic mosaic pattern around the tumor. **c** Microcalcifications of various sizes around the tumor were detected in ultrasound (*arrows*)

**Fig. 2:**
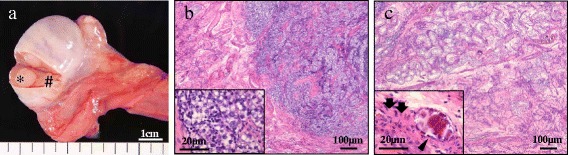
**a** Macroscopic findings of orchiectomy specimen. A tiny yellowish tumor was observed (*), surrounded by normal tissue (#). **b** Microscopic findings of the tiny tumor. Pathological findings revealed a pure seminoma. **c** Microscopic findings of the tissue around the tumor: calcification lesions (*black arrowhead*) and fibrous structures (*black arrows*)

**Fig. 3:**
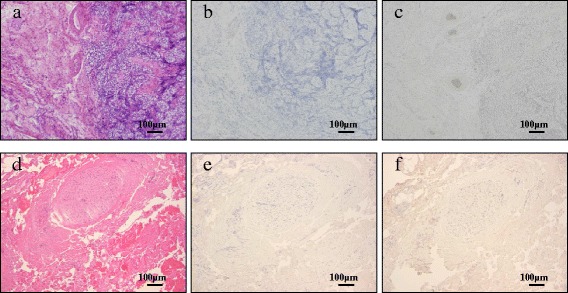
Hematoxylin and eosin and immunohistochemical staining of the orchiectomy specimen (**a, b, c**) and of the retroperitoneal dissected specimen (**d, e, f**). **b** and **e** alpha-fetoprotein staining; **c** and **f** DUPAN-2 staining

A 29-year-old Asian man with no remarkable past history was referred to our hospital presenting with right flank pain. He had an episode of right flank pain 2 weeks before the first visit. Computed tomography (CT) revealed a retroperitoneal mass (52×36×31 mm) located between his aorta and inferior vena cava (Fig. 1a), but a testicular tumor was not detected in his testis. His serum levels of LDH, AFP, and DUPAN-2 were high (327 U/l, 29.6 ng/ml, and higher than measurable range, 1600 U/ml, respectively). Serum levels of other tumor markers, such as hCG, carcinoembryonic antigen, carbohydrate antigen 19–9, and soluble interleukin 2 receptor, were within the normal range (1.1 mIU/ml, 2.7 ng/ml, 27.9 U/ml, and 157 U/ml, respectively). Although no tumor or nodule was palpable in either testis, ultrasonography revealed multiple low echoic lesions in his right testicular parenchyma (Fig. 1b). He was diagnosed with right testicular cancer with retroperitoneal metastasis, and underwent right high orchiectomy (Fig. 2a). A pathological examination revealed pure seminoma (Fig. 2b); fibrous foci were diffusely observed (Fig. 2c) and no nonseminomatous components were found in the specimen. Because his preoperative serum AFP levels were high, induction chemotherapy combining cisplatin, etoposide, and bleomycin (BEP), was performed. After three courses, his serum AFP and DUPAN-2 levels were normalized. The volume of his retroperitoneal mass decreased to 15×10×7 mm after three additional courses of chemotherapy combining etoposide and cisplatin (EP). Finally, laparoscopic retroperitoneal lymph node dissection (RPLND) was performed, and pathology of the dissected specimen showed fibrous and necrotic tissue with no viable cells (Fig. 3d). He is alive without recurrence 54 months after orchiectomy.

## Discussion

Patients with elevated serum AFP levels and a primary pure seminoma are treated as patients with NSGCTs, because some nonseminomatous components are presumed to be present either in the testis or at a metastatic site. Therefore, we treated the current case as NSGCTs, and induction chemotherapy followed by RPLND was performed after orchiectomy as in stage IIB NSGCTs. Peterson *et al*. [5] reported 42 cases with pure seminoma at orchiectomy and showing elevated serum AFP levels. They performed combination chemotherapy based on platinum reagent followed by RPLND. Viable cancer cells were observed in the retroperitoneal lymph nodes of 15 patients (37.5 %) with pure seminoma showing elevated serum AFP levels. In general, 16.9 % of patients with stage II or greater pure seminoma without elevated serum AFP levels have viable cancer cells in their retroperitoneal lymph nodes [6]; of the patients with NSGCTs who have retroperitoneal lymph node metastases, 12.2 % have viable cancer cells in their retroperitoneal lymph nodes after chemotherapy [7]. Therefore, pure seminomas with elevated serum AFP levels have a relatively lower sensitivity to chemotherapy than pure seminomas without elevated serum AFP levels or NSGCTs. Fortunately, the pathological examination of the retroperitoneal lymph nodes in our patient did not reveal viable cancer cells; the long-term good prognosis suggests that six courses of BEP and EP chemotherapy were effective in this patient.

DUPAN-2 is a well-known tumor marker for pancreatic, bile duct, and colorectal cancers [8]; in our case, serum DUPAN-2 levels, as well as AFP, were increased before orchiectomy. In the literature, only two cases of embryonal carcinomas with elevated serum DUPAN-2 levels have been reported [9, 10]. Kamoshida *et al*. reported immunohistochemical analyses of DUPAN-2 expression using paraffin-embedded specimens of 30 testicular germ cell tumors, and DUPAN-2 was expressed in all of the specimens from nine patients with embryonal carcinoma [11]. In our case, before orchiectomy, our patient’s DUPAN-2 level was higher than the measurable upper limit and it decreased to within the normal range after chemotherapy induction, suggesting that DUPAN-2 in our patient was produced by a nonseminomatous component like an embryonal carcinoma.

Seminomas typically appear as solid, round, homogeneous, low reflective masses contained within testes, without calcification inside the tumors. On the other hand, embryonal carcinomas and teratomas appear as inhomogeneous masses with calcification inside the tumor mass and, generally, a normal gonadal stroma does not contain calcifications. Miller *et al*. reviewed 3854 testicular ultrasound examinations; they hypothesized that intratesticular microcalcifications outside the margin of the tumor might represent a phenomenon of burned-out tumor [12]. In our case, an ultrasound examination revealed rough microcalcifications around the intratesticular tumor; in the orchiectomy specimen (Fig. 1c), many calcifications and fibrous tissues were detected around the seminomatous tumor (Fig. 2c). Those findings suggest that the testicular mass might result from a burned-out phenomenon of an embryonal carcinoma. Therefore, we performed immunohistochemical analyses for DUPAN-2 and AFP on the orchiectomy specimen and retroperitoneal mass. Immunohistochemical analyses showed no positive cells for DUPAN-2 or AFP in the orchiectomy specimen and retroperitoneal mass (Fig. 3). If we had obtained a biopsied retroperitoneal specimen in the first diagnosis, we might have been able to detect positive cancer cells.

In conclusion, here we report a case of pure testicular seminoma with elevated serum AFP and DUPAN-2 presenting retroperitoneal metastasis. We recommend an ultrasound examination of bilateral testes when large retroperitoneal tumors are detected in young men, even if a mass is not palpable in the scrotum.

## Conclusions

We report a case of pure testicular seminoma at orchiectomy with elevated serum AFP and DUPAN-2 presenting retroperitoneal metastasis. When a retroperitoneal mass is detected and a testicular tumor is not palpable, we recommend consideration of the possibility of a testicular tiny tumor or of a burned-out phenomenon.

## Consent

Written informed consent was obtained from the patient for publication of this case report and any accompanying images. A copy of the written consent is available for review by the Editor-in Chief of this journal.

## Abbreviations

AFP: alpha-fetoprotein
BEP: cisplatin, etoposide, and bleomycin
CT: computed tomography
EP: etoposide and cisplatin
hCG: human chorionic gonadotropin
LDH: lactate dehydrogenase
NSGCTs: non-seminomatous germ cell tumors
RPLND: retroperitoneal lymph node dissection
